# Investigations of Intraoral Scan Body Extensions. Effect of Design on the Accuracy, Usability, and Feasibility for Digital Full‐Arch Implant Scans In Vitro

**DOI:** 10.1111/clr.70158

**Published:** 2026-07-17

**Authors:** Julian Hessel Baranowski, Michael Braian, Victoria Franke Stenport, Ann Wennerberg

**Affiliations:** ^1^ Department of Prosthodontics/Dental Materials Science, The Sahlgrenska Academy University of Gothenburg Gothenburg Sweden; ^2^ Center for Oral Rehabilitation Public Dental Service Östergötland Linköping Sweden; ^3^ Swedish Organisation for Computer Aided Digital Dentistry SWECADD Malmö Sweden; ^4^ The Brånemark Clinic Gothenburg Sweden

**Keywords:** computer‐aided design, dental implants, dental impression technique, dental prosthesis, dimensional measurement accuracy, edentulous, implant‐supported, jaw, printing, three‐dimensional

## Abstract

**Objectives:**

To evaluate the effect of different scan body extension designs on the accuracy, usability, and feasibility of digital full‐arch implant impressions in an edentulous mandibular model. Accuracy was expressed as trueness and precision, usability as scan time, and feasibility as the number of total scan failures.

**Material and Methods:**

An edentulous mandibular model with four implants was fabricated with minimal surface morphology. Five scan body extension configurations were tested: no extensions (control), commercially available extensions, conventional impression copings used as extensions, and two morphological 3D‐printed extensions oriented between adjacent implants or toward the center of the arch. Two intraoral scanners were used. For each scanner and configuration, 30 scans were acquired. Inter‐implant distances were compared against coordinate measuring machine reference values. Trueness was assessed as mean absolute deviation, precision as standard deviation, and differences were analyzed with non‐parametric tests.

**Results:**

The highly morphological scan body extensions produced the most consistent improvements. Combined across scanners, trueness improved by 40–57 μm relative to control for the longest inter‐implant span. Precision improved significantly for the center‐oriented configuration. All scan body extension techniques reduced scan time by 18%–41% and reduced scan failures from 13% (control) to 3% for the morphological designs. Improvements were most pronounced for longer spans and varied between scanners.

**Conclusions:**

Highly morphological 3D‐printed scan body extensions significantly improved the accuracy, usability, and feasibility of complete‐arch digital implant impressions compared to an unmodified control workflow. Clinical validation is required to confirm these findings for broader use.

## 1 Introduction

Achieving an accurate fit for multi‐unit, implant‐supported prostheses remains a central endeavor in prosthetic dentistry in order to limit misfit at the mating surfaces in the framework‐implant complex. Even a modest misfit can translate into stress within retaining screws, abutments, and frameworks, and may compromise long‐term performance when errors accumulate across longer spans (Abduo et al. 2010; Nogueira et al. 2018; Pan et al. 2021).

Current literature indicates that clinically observable marginal misfits of suprastructures are quite challenging to detect and there is still no consensus on a clinically acceptable misfit threshold. Suggested thresholds range from 100 to 150 μm, though these values remain empirical and lack strong scientific validation. However, there is a general agreement that the ambition to achieve a lower misfit in all steps of the treatment is preferable, which underscores the need to prioritize accuracy even at the impression step (Abduo and Lyons 2012; Andriessen et al. 2014; Katsoulis et al. 2017; Pan et al. 2021).

Furthermore, digital intraoral scanning (IOS) offers workflow efficiency and patient comfort and is increasingly used for tooth‐supported fixed dental prostheses (FDPs) and short‐span implant cases. However, the outcomes from scientific reports are conflicting when digital and conventional impressions are compared at the full‐arch level (Ahlholm et al. 2018). A randomized clinical trial in maxillary full arches found digital impressions clinically and radiographically acceptable and significantly faster than conventional pick‐up (Cappare et al. 2019). Broader in vitro reviews still caution that long spans and multiple implants remain challenging to achieve the same level of accuracy and precision due to stitching errors, pointing to the immaturity of digital impressions in edentulous complete‐arch implant treatments (Orejas‐Perez et al. 2022).

A principal factor contributing to reduced accuracy in completely edentulous arches is the lack of stable anatomic landmarks between scan bodies. When surface morphology is sparse, continuous stitching becomes error‐prone, leading to distance and angular deviations and, in some cases, total scan failures (Kernen et al. 2023). Early clinical work in edentulous mandibles has documented stitching problems and unacceptable inter‐implant distance deviations (Andriessen et al. 2014). Subsequent in vivo and in vitro studies confirmed that errors increase with inter‐implant distance, and that scanner technology and scan‐body design further modulate outcomes (Mizumoto et al. 2020; Orejas‐Perez et al. 2022). Consequently, both trueness (proximity to true physical values) and precision (repeatability of the 3D position values between subsequent scans) must be assessed explicitly when evaluating complete‐arch protocols.

Several strategies have been proposed to mitigate landmark scarcity in edentulous spans: splinting scan bodies with resin or floss, adding surface markers or pastes to soft tissue, or using custom auxiliary devices (scan body extensions) (Bader Eddin and Önöral 2024; Imburgia et al. 2020; Mizumoto et al. 2020; Tallarico et al. 2020). Another strategy involves stereophotogrammetry to complement intraoral scanning, though this introduces additional treatment steps and costs (Orejas‐Perez et al. 2022).

In vitro evidence indicates that scan body extensions can improve trueness, sometimes markedly, reduce scan failures, and in some implementations yield accuracy comparable to conventional splinted open‐tray impressions (Azevedo et al. 2025; Fu et al. 2025). Thus, scan body extensions represent a clinically appealing strategy as they do not introduce additional treatment steps or major costs.

Despite previous promising results, prior work suggests that stitching accuracy depends both on the scanner model and on the use of scan body extensions. The scan body extensions evaluated in most studies have featured relatively simple geometric shapes with limited morphology (Azevedo et al. 2025; Bader Eddin and Önöral 2024; Fu et al. 2025). However, Kernen et al. (2022) showed that an irregular scan body extension design improved trueness compared to circular or square alternatives, suggesting that increased geometric complexity may benefit stitching accuracy. Building on this observation, the present study investigated whether more elaborate morphology could yield further improvements. The custom scan body extensions were therefore designed with tooth‐like features to mimic the surface characteristics that intraoral scanners are optimized to capture. The restricted designs in previous studies are likely related to manufacturing challenges, but with the widespread availability of stereolithography (SLA) 3D printers, more complex morphologic designs are now feasible to produce chairside or by a cooperating dental laboratory. A further goal was to develop scan body extensions that could be easily self‐produced and rigidly connected to existing scan bodies without requiring a prior impression or additional patient visit.

The working hypothesis of this study was that highly morphological scan body extension designs would improve accuracy, usability and feasibility of full‐arch digital implant impressions compared to simpler geometries or unmodified control workflows, and that performance would differ between scanner systems. The aim was to evaluate and compare four scan body extension techniques: a commercially available 3D‐printed system, conventional impression copings used as scan body extensions, and highly morphological 3D‐printed scan body extensions in two different positions, against an unmodified control workflow, tested across two intraoral scanner systems.

## 2 Material and Methods

### 2.1 Reference Mandibular Model and Implant Configuration

An edentulous mandibular arch was digitally designed with extremely limited surface morphology to reproduce a highly challenging clinical scenario for intraoral scanning. The model was additively manufactured with a stereolithography (SLA) printer (Form 4B, Formlabs Inc., Somerville, MA, USA) using a denture base resin (Denture Base Resin, Formlabs Inc., Somerville, MA, USA), according to the manufacturer's recommendations.

Four internal‐connection dental implants with varied angulation (Neoss ProActive Straight SP, Neoss, Gothenburg, Sweden) were designed within a digital model to represent a typical all‐on‐four configuration (Tsigarida and Chochlidakis 2021). After printing, the corresponding implant analogs were permanently embedded in the model at the planned positions and labeled P1–P4, with P1 being the most posterior position in quadrant four and P4 the most posterior position in quadrant three. The inter‐implant distances were approximately 18 mm (P1–P2), 34 mm (P1–P3), and 42 mm (P1–P4) (Figure 1).

**FIGURE 1:**
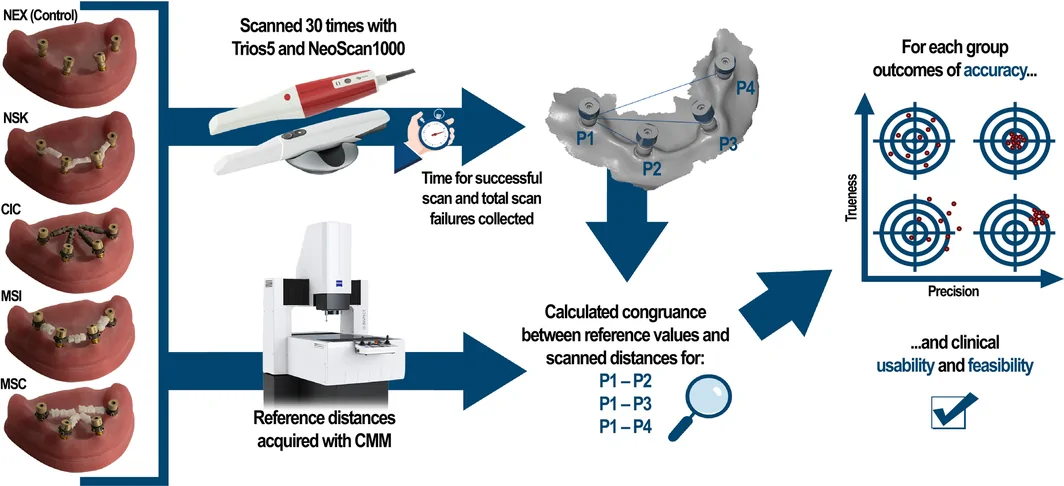
Illustration of the workflow.

On each implant, a straight multi‐unit abutment (Neoss Multi‐Unit Abutment 1 mm, 0°, Neoss, Gothenburg, Sweden) was mounted and torqued to 25 Ncm according to the protocol of the manufacturer to simulate a clinical situation where the impression was made at abutment level. Titanium multi‐unit scan bodies (Scan Body Ti, Neoss Gothenburg, Sweden) were then mounted on the abutments and torqued to 25 Ncm to minimize the risk of displacement during scanning.

### 2.2 Scan Body Extension Design and Fabrication

#### 2.2.1 Readily Available Scan Body Extensions

Prefabricated scan body extensions (ScanKey, Neoss, Gothenburg, Sweden) were used as a commercially available auxiliary device. In accordance with the manufacturer's recommendations, the devices were 3D printed, using an SLA printer and resin (Form 3, Formlabs Inc.; White Resin V4.1, Formlabs Inc., Somerville, MA, USA). The manufacturer does not specify a particular resin beyond the requirement that it produces an opaque, non‐reflective surface suitable for optical scanning, and lists Sprintray Study Model White 2 as an example of a compatible material; the White Resin V4.1 used in this study fulfills the same optical criteria. Post‐processing followed the manufacturer's protocol: parts were immersed in 99% isopropanol for approximately 2 min, allowed to dry for 10 min, and then light‐cured for 30 min. This process produced a rigid snap‐on fit onto the scan bodies.

#### 2.2.2 Titanium Mounting Rings and Conventional Impression Copings

To allow secure fixation of custom scan body extensions and impression copings, four titanium mounting rings were designed (CAD) with a central bore matching the scan body and four radial M1.6 × 0.35 mm threaded holes oriented at 0°, 10°, 20°, and 30° relative to the horizontal direction of the scan body top surface. The rings were manufactured by a microtechnology company (Kullbergs Mikroteknik AB, Lycke, Sweden) and were designed to be fastened to the scan bodies with titanium screws (Neo Laboratory Screw NP, Neoss, Gothenburg, Sweden), thereby reducing the risk of unintended dislodgement and complete scan failure during IOS acquisition (Figure 2).

**FIGURE 2:**
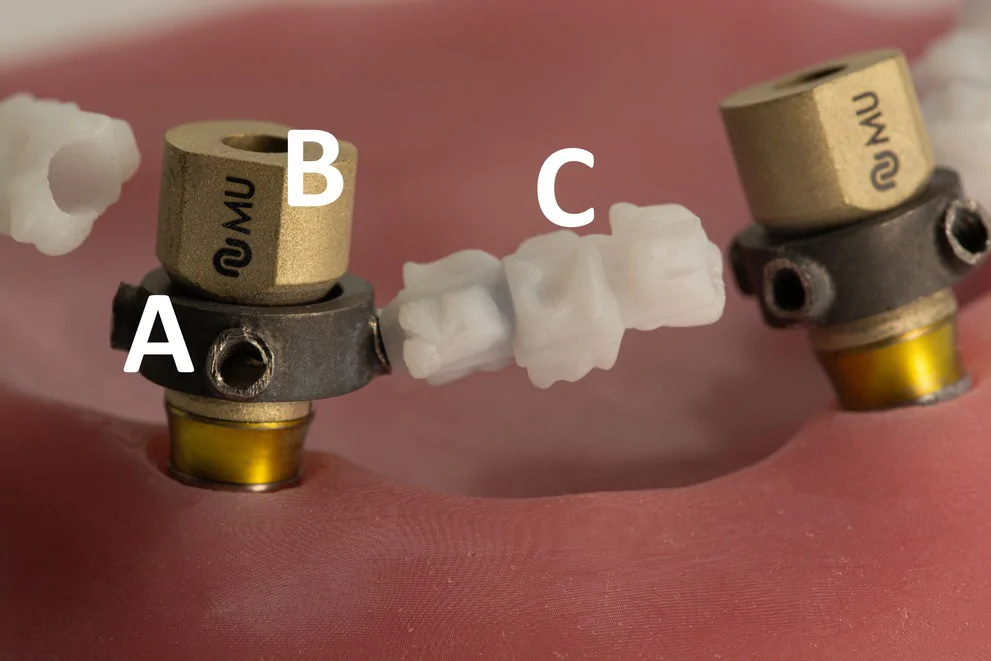
Close up image of the (A) titanium mounting rings, (B) scan body, (C) morphologic scan body extension mounted on an abutment level scan body.

The internal threading of the rings was made identical to that of conventional pick‐up impression copings (M1.6). Consequently, conventional impression copings (Neoss, Gothenburg, Sweden) were mounted onto the rings and used as scan body extensions in one of the experimental groups. To reduce surface reflectivity and facilitate scanning, the impression copings were air‐abraded with 50 μm aluminum oxide at approximately 1 bar pressure and ultrasonically cleaned prior to use.

#### 2.2.3 Custom Made 3D‐Printed Morphological Scan Body Extensions

Custom‐made scan body extensions were digitally designed (CAD) for the study to introduce an additional, highly distinctive morphology into the edentulous arch. The extensions consisted of small tooth‐like features with randomized shape and arrangement at approximately 1 mm scale to enhance the ability of IOS algorithms to track and stitch sequential images. A central screw channel was incorporated to allow for fixation using an M1.6 screw (Neo Laboratory Screw NP Neoss, Gothenburg, Sweden), enabling rigid fixation of the scan body extension onto the titanium ring while leaving the essential scan body geometry accessible for subsequent CAD alignment (Figure 2).

Two lengths of scan body extensions (short and long) were created to accommodate different inter‐implant distances, and four random shape variations were generated to reduce the risk of the IOS software mistaking one extension for another. The scan body extensions were printed in White Resin V4.1 on a Form 3 printer (Formlabs Inc., Somerville, MA, USA).

For one configuration the custom scan body extensions were oriented to bridge toward the next nearest implant, effectively connecting adjacent scan bodies along the arch (Figure 2). For the other configuration (morphological scan body extensions toward center—MSC), all scan body extensions were oriented toward the center of the arch to create a compact cluster of morphology and minimize the effective span between the first and last implant by following the shortest geometric path.

### 2.3 Experimental Groups

Five groups were defined according to the scan body extension configuration; see Figure 3.

**FIGURE 3:**
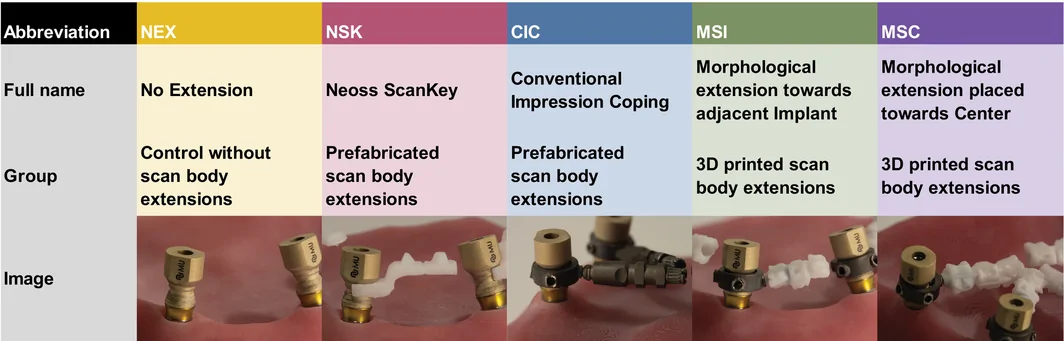
Overview of groups and their abbreviations.

### 2.4 Scanning Procedure

All scans were performed by a single operator with several years of IOS experience in a temperature‐ and light‐controlled environment (24°C; ambient illuminance < 1000 lx). The mandibular model was rigidly fixated for all recordings.

Two IOS devices were evaluated:

NeoScan1000 (abbreviated NS), using NeoConnect version 1.0.12.1 (Neoss, Gothenburg, Sweden).

TRIOS 5 (abbreviated T5), using 3Shape App 24.3.1/R1.03 configuration 3.27.2.1. Dental Desktop 7.83.0.2 (3Shape A/S, Copenhagen, Denmark). The AI support function was disabled for all TRIOS 5 acquisitions, as this feature has been shown to distort surface scan results, particularly in complete‐arch implant scans (Revilla‐León et al. 2025).

Before recording data for each combination of scanner and group, four consecutive practice scans were performed to familiarize the operator with the specific scan body extension configuration and minimize learning effects. The training scans were not included in the analysis.

For each group and IOS, 30 consecutive successful scans of the complete arch were obtained, resulting in a target of five groups × 2 IOSs × 30 scans = 300 experimental datasets. To minimize systematic bias from the scanning sequence, the order in which the IOSs were used was alternated between groups: Group 1 was scanned first with NS and then T5; Group 2 with T5 then NS; Group 3 with NS then T5, and so on.

All scans were acquired using the complete‐arch edentulous implant scanning protocol recommended by each IOS manufacturer, starting from the most posterior implant in quadrant four (P1, Figure 1) and proceeding along the arch. Both scanners followed a two‐stage protocol: a pre‐operative arch scan was first acquired, after which the scan data at each implant position was removed and scanning was repeated with the scan bodies in place. To avoid the confounding effect of repeated scan body reattachment between scans, the scan bodies and extensions were present during the pre‐operative scan as well, which represents a deviation from the clinical workflow where the pre‐operative scan is typically acquired before scan body placement. An occlusal‐vestibular technique was used for all groups: the operator first completed an occlusal pass along the full arch, followed by buccal and lingual passes. The same scanning movement was used for both IOS devices. For the center‐oriented configurations (MSC and CIC), a supplementary occlusal pass over the centrally positioned extensions was performed after the initial arch pass, followed by the lingual and buccal infill of the edentulous ridge between scan bodies.

The extensions were generally reproduced in the buccal and lingual aspects, as their surface morphology aided acquisition of the edentulous ridge. However, complete reproduction from all angles, including the underside of the extensions, was not specifically targeted, as only intact scan body geometry would be required for subsequent CAD alignment and therefore considered a successful scan.

For each scan attempt, the timer integrated in the IOS software was started at the first image capture and stopped when the scan was accepted as complete with no obvious stitching defects and all four scan bodies adequately reproduced. If major non‐recoverable stitching errors occurred (e.g., duplicated or missing scan bodies), the attempt was classified as a total failure and discarded. A new scan was initiated until 30 valid scans had been obtained for that group and IOS. The number of failed attempts and the scan time (s) for each successful scan were recorded.

### 2.5 Reference Measurements: Laboratory Scanner and Coordinate Measuring Machine (CMM)

After completion of Groups 1 and 2, the mandibular model was digitized with a laboratory scanner (E3 using ScanIt Dental v.2.2.5.0, 3Shape A/S, Copenhagen, Denmark) to obtain an initial high‐resolution reference dataset. The scan‐bodies were removed and reattached in the same positions with the metal rings to allow attachment of further scan body extensions. Following completion of all IOS acquisitions (Groups 3–5), the model was scanned again with the same laboratory scanner to assess any potential changes in scan body positions related to reattachment between groups.

The model was then measured with a coordinate measuring machine (CMM) (O‐Inspect, Carl Zeiss AG, Oberkochen, Germany) using dedicated software (Calypso version 7.4.20, Carl Zeiss AG, Oberkochen, Germany). On each scan body, a best‐fit cylinder was constructed and a center point at the level of the mating surface with the multi‐unit abutment was calculated and named Point 1–4 (P1–P4). The Euclidean distances between P1 and the other points (P1–P2, P1–P3, P1–P4) were measured and used as reference values for subsequent deviation analysis.

### 2.6 Deviation Analysis

All IOS datasets (STL format) were imported into a metrology software (GOM Inspect 2018 Hotfix 5, Rev.115656, Build 2019‐02‐15; GOM Metrology, Braunschweig, Germany). For each scan, virtual cylinders were fitted to the surfaces corresponding to the four scan bodies at the level of the abutment interface, replicating the CMM protocol. The center points of these cylinders were extracted, and the three‐dimensional Euclidean distances between P1 and each of P2, P3, and P4 were calculated.

For each inter‐implant span, the linear absolute deviation Δ*d* was defined as follows:

$$\mathrm{\Delta d}=\sqrt{\left({\left(\text{distance}\;\mathrm{IOS}-\text{distance}\;\mathrm{CMM}\right)}^{2}\right)}$$

where distance IOS is the distance measured from the IOS dataset and distance CMM is the corresponding reference distance from the CMM. Positive values would indicate an overestimation of the span, and negative values an underestimation. To quantify trueness, absolute deviations were used for statistics.

Trueness was evaluated for each scanner–group combination as the mean absolute deviation per span. Precision was evaluated as the standard deviation (SD) of repeated measurements within each scanner–group combination.

### 2.7 Statistical Analysis

Statistical analysis was performed with a statistical software package (SPSS Statistics, version 31.0, IBM Corp., Armonk, NY, USA). For each IOS and scan body extension configuration, descriptive statistics were calculated for the absolute deviations of individual spans, scan time, and number of total failures.

A normality test was performed for the deviation and scan‐time data which showed that normality of data cannot be assumed for both (Kolmogorov–Smirnov).

For trueness, the Kruskal–Wallis test was chosen with scan body extension configurations (NEX, NSK, CIC, MSI, MSC) and Mann–Whitney for the scanner (T5, NS); post hoc pairwise comparisons for the scan body extensions were adjusted using Bonferroni correction. Separate models were also run for each inter‐implant span (P1–P2, P1–P3, P1–P4) to assess whether errors accumulated with increasing distance.

Precision was analyzed by comparing the dispersion of measurements within groups. Variances of deviations for each scan body extension configuration were compared with the control (NEX) using Levene's test. To account for multiple pairwise variance comparisons (four scan body extension groups vs. control), the significance level was adjusted with Bonferroni correction (*α* adjusted = 0.05/4≈0.0125).

### 2.8 Outcome Variables

The primary outcome was accuracy, expressed as trueness (mean absolute deviations from reference values) and precision (standard deviation of repeated measurements). Secondary outcomes were usability, assessed by scan time, and feasibility, assessed by the number of scan failures due to major stitching errors. The null hypotheses were: (1) no significant differences in trueness or precision between scan body extension groups and control, (2) no significant differences between the two intraoral scanners, and (3) no significant differences in scan time or scan failure rate among scan body extension groups compared to control.

## 3 Results

### 3.1 Effect of Inter‐Implant Distance on Accuracy

Comparison of the two laboratory scanner datasets, acquired before and after reattachment of the scan bodies and mounting rings, revealed differences ranging from 12 to 24 μm at the three implant positions.

Analysis of shorter inter‐implant spans revealed similar patterns considering trueness and precision to those observed for the longest span (P1–P4) (Table 1), although with varying magnitudes of effect (Tables S3 and S4).

**TABLE 1:** Descriptive statistics of absolute linear distance deviations (P1–P4) for trueness and precision across scan body extension groups and scanners.

Scan body extension group	Scanner	Across scan body extension groups split by scanner					Across scan body extension groups				
		*n*	Trueness		Precision		*n*	Trueness		Precision	
			Mean (μm)	*p*	SD (μm)	*p*		Mean (μm)	*p*	SD (μm)	*p*
NEX	Neoscan1000	30	180	—	±53	—	60	143	—	±72	—
	Trios5	30	106	—	±71	—					
NSK	Neoscan1000	30	136	< 0.001**	±94	0.045	60	128	0.03	±105	0.171
	Trios5	30	120	0.69	±116	0.135					
CIC	Neoscan1000	30	170	0.54	±32	0.011***	60	116	0.05	±66	0.634
	Trios5	30	61	0.008**	±42	0.027					
MSI	Neoscan1000	30	127	< 0.001**	±61	0.538	60	86	< 0.001**	±64	0.378
	Trios5	30	45	< 0.001**	±34	0.005***					
MSC	Neoscan1000	30	143	0.002**	±27	0.002***	60	103	0.004**	±48	< 0.001***
	Trios5	30	63	0.04	±26	< 0.001***					
Total	Neoscan1000	150	152	< 0.001*	±61	< 0.001*	300	115	< 0.001*	±76	< 0.001*
	Trios5	150	79	< 0.001*	±72	< 0.001*					

*Note:* Lower values represent higher accuracy.

Abbreviations: CIC, conventional impression copings used as scan body extensions; MSC, morphological scan body extensions positioned toward center of the arch; MSI, morphological scan body extensions positioned toward adjacent implant; NEX, control group without scan body extensions; NSK, Neoss ScanKey scan body extension.

* *p* < 0.05: Significant difference between scanners (Mann–Whitney *U*) or between scan body extension groups (Kruskal–Wallis).

** *p* < 0.0125: Significant post‐hoc difference from control (Kruskal–Wallis with Bonferroni adjustment).

*** *p* < 0.0125*:* Significant variance difference from control (pairwise Levene's tests with Bonferroni correction).

For the intermediate span (P1–P3), trueness values across all scan body extension groups ranged from 82 to 108 μm, compared to 122 μm for the control. The morphological scan body extensions (MSI and MSC) and conventional impression copings (CIC) demonstrated statistically significant improvements of 25% to 33% when scanners were combined, with Trios5 consistently achieving lower deviations (41–78 μm) than NeoScan1000 (104–139 μm). Precision for P1–P3 showed a statistically significant improvement of 18 μm only for the MSC configuration (35% improvement, *p* < 0.001).

For the shortest span (P1–P2), absolute deviations were generally lower, ranging from 47 to 69 μm across scan body extension groups compared to 67 μm for control. None of the scan body extension configurations yielded statistically significant improvements in trueness when scanners were combined; however, when evaluated separately, statistically significant improvements were observed for MSC, MSI, and CIC with NeoScan1000, and for MSI and CIC with Trios5. Precision improvements for P1–P2 were statistically significant for all scan body extension groups except NSK (*p* < 0.001 for CIC, MSI, and MSC).

Across all three spans, errors tended to increase with increased inter‐implant distance (Figure 4), and the benefit of highly morphological scan body extensions was most pronounced for longer spans and when using Trios5 (Figure 5).

**FIGURE 4:**
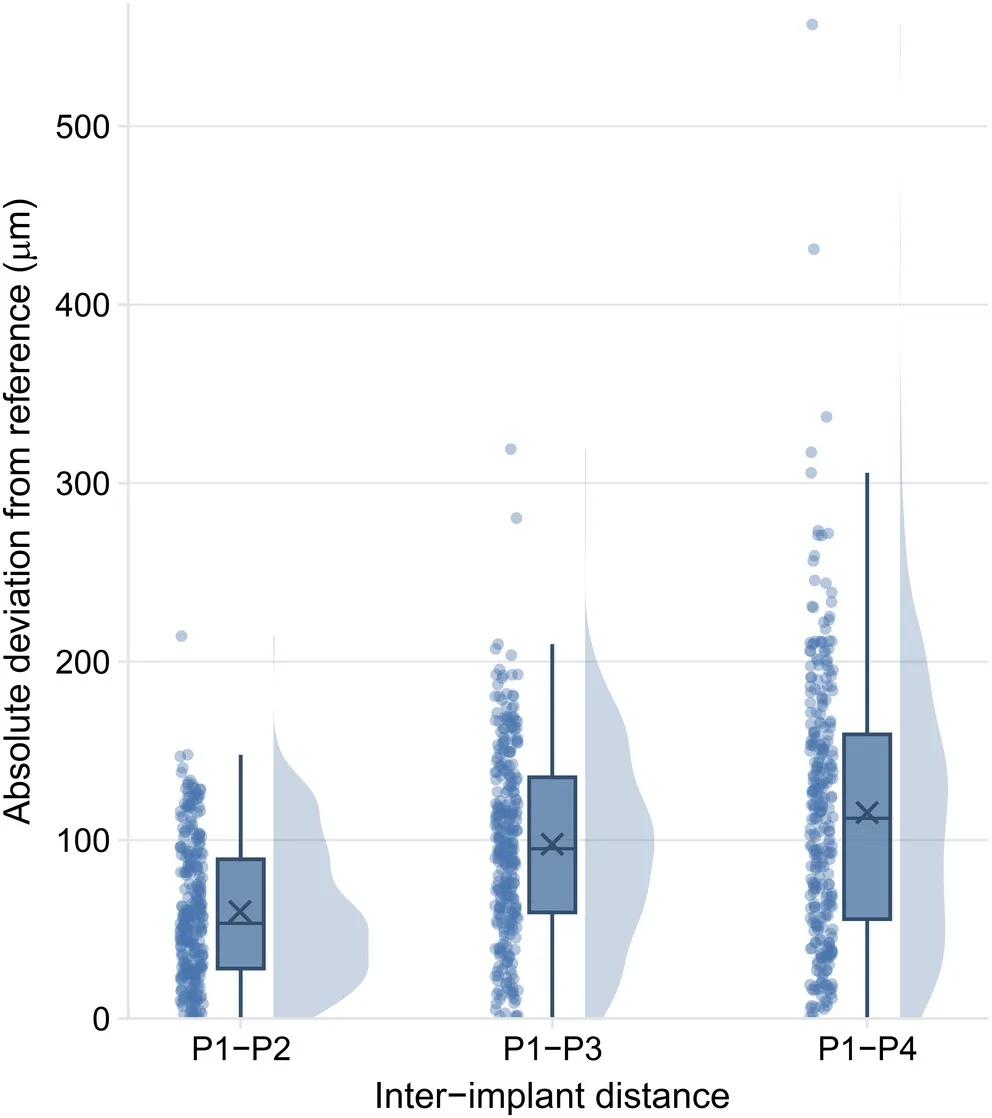
Graph illustrates the effect of inter‐implant distance on trueness (mean values) and precision (variability) across all groups and scanners. For visual reference of the inter‐implant distances, see Figure 1.

**FIGURE 5:**
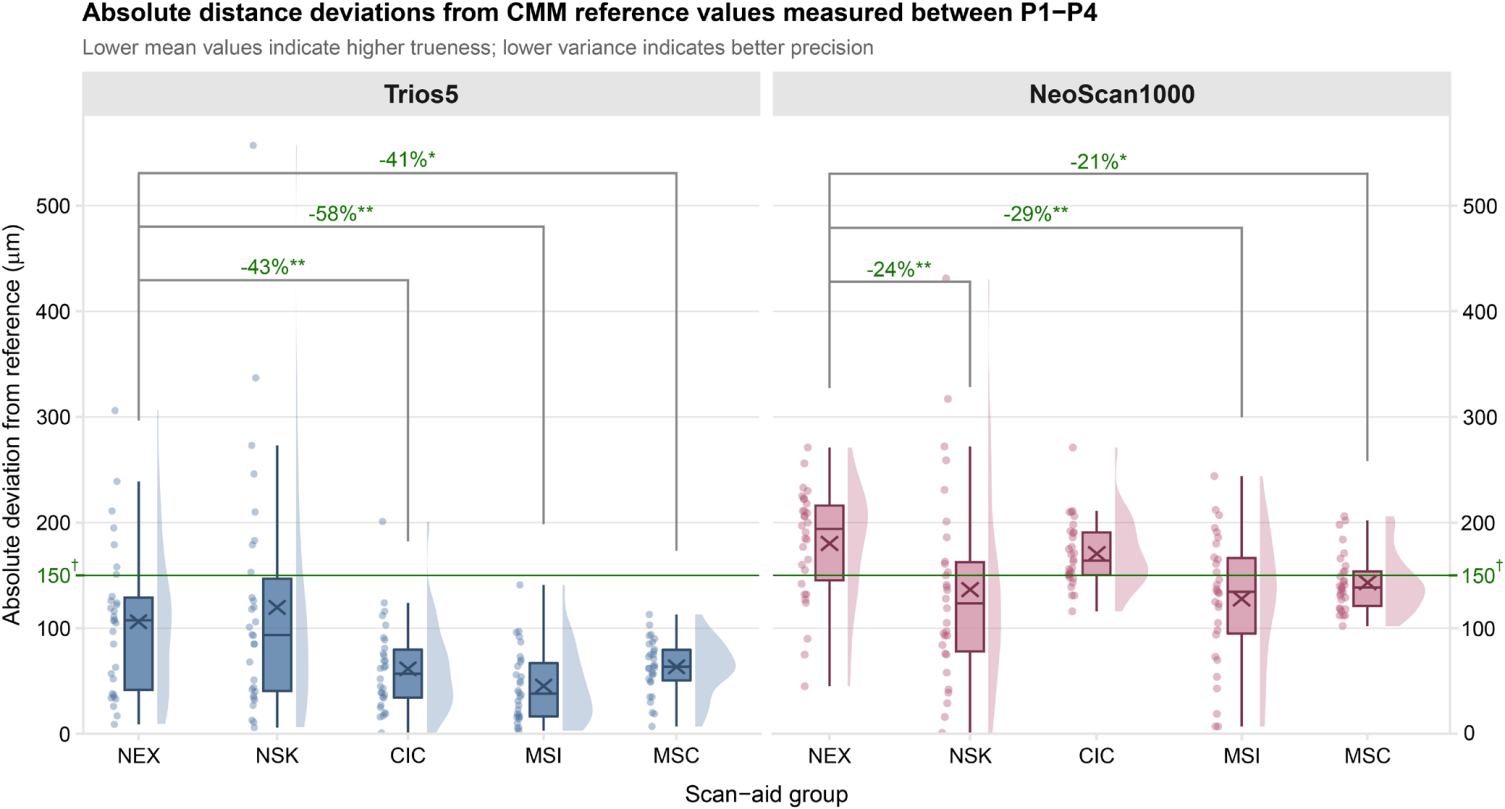
Illustration of absolute distance deviations for each scan body extension group and scanner type. †, clinically suggested misfit threshold; CIC, conventional impression copings used as scan body extensions; MSC, morphological scan body extensions positioned toward center of the arch; MSI, morphological scan body extensions positioned toward next implant; NEX, control group without scan body extensions; NSK, Neoss ScanKey scan body extension.

### 3.2 Trueness

Relative to the unmodified control workflow (NEX), a majority of supplementary scan body extension approaches demonstrated a directionally improved trueness, although the magnitude of improvement varied between techniques and scanners. The differences in trueness were most pronounced for the inter‐implant distance, P1–P4 (Table 1); therefore, subsequent trueness results are reported primarily for this distance (Figure 5). Results for P1–P2 and P1–P3 are reported in Tables S3 and S4.

For the NSK prefabricated scan body extension, no statistically significant improvement in trueness was observed when data from both scanners were combined. When examined separately, a statistically significant improvement relative to the control was observed with NeoScan1000 (120 μm vs. 180 μm), whereas no relevant difference was detected with Trios5.

The CIC approach, using conventional impression copings as scan body extensions, likewise showed no significant trueness improvement relative to the control when scanners were combined. Individually, NeoScan1000 demonstrated only minimal and non‐significant differences, while Trios5 showed a statistically significant improvement compared to the control (61 μm vs. 106 μm).

Both 3D‐printed highly morphological scan body extension configurations (MSI and MSC) demonstrated the most consistent improvements in trueness. When scanners were combined, trueness improved by approximately 40–57 μm relative to the control, with the inter‐implant design (MSI) showing the most consistent reduction in deviations; these improvements were statistically significant for both configurations. Scanner‐specific analysis showed improvements of 53 μm (29%) for MSI and 37 μm (16%) for MSC with NeoScan1000, whereas Trios5 demonstrated more pronounced improvements of 61 μm (58%) and 43 μm (41%), respectively.

Across all scans, trueness differed significantly between scanners. Trios5 showed lower absolute deviations (mean 79 μm, range 1 to 337 μm) compared to NeoScan1000 (mean 152 μm, range 0.8 to 557 μm).

### 3.3 Precision

Precision analysis revealed a broadly similar pattern to that observed for trueness, with improved precision for a majority of the scan body extension designs compared with the control (Table 1). As for trueness, the largest precision differences were observed for the P1–P4 inter‐implant distance, and subsequent precision results are therefore reported for this distance (Figure 5).

For the NSK scan body extension, precision deteriorated relative to the control when data from both scanners were combined, corresponding to an increase in variability of 33 μm (46%); however, this difference was not statistically significant. When evaluated separately, no scanner showed a statistically significant difference in precision compared to the control.

The CIC and MSI techniques demonstrated precision values comparable to the control when scanners were compared, demonstrating statistically insignificant differences of 10% as compared to the control group. When assessed individually, inconsistent results for precision were observed, with a statistically significant improvement of 21 μm (40%) for CIC with NeoScan1000 and 37 μm (52%) for MSI with Trios5.

The MSC scan body extension showed more consistent results, with a statistically significant improvement in precision of 24 μm (33%) relative to the control when scanners were combined. Corresponding improvements of 26 μm (49%) for NeoScan1000 and 45 μm (63%) for Trios5 were observed when the scanners were considered separately.

Overall, precision outcomes were more strongly influenced by scan body extension design than by scanner type, with no statistically significant differences detected between scanners but statistically significant differences observed across scan body extension groups.

### 3.4 Time to Complete Scan

All supplementary scan body extension techniques reduced scanning time relative to the control group (Table 2).

**TABLE 2:** Descriptive statistics of scanning time for successful scans and number of total scan failures.

Group	Scanner	Across scan body extension groups split by scanner				Across scan body extension groups			
		*n*	Time (s)	*p*	*n* of scan failures	*n*	Time (s)	*p*	*n* of scan failures
NEX	Neoscan1000	30	112 ± 14	—	4 (13%)	60	121 ± 17	—	8 (13%)
	Trios5	30	131 ± 15	—	4 (13%)				
NSK	Neoscan1000	30	89 ± 15	< 0.001**	6 (20%)	60	99 ± 19	< 0.001**	17 (28%)
	Trios5	30	110 ± 17	0.019	11 (37%)				
CIC	Neoscan1000	30	79 ± 14	< 0.001**	3 (10%)	60	85 ± 14	< 0.001**	8 (13%)
	Trios5	30	90 ± 13	< 0.001**	5 (17%)				
MSI	Neoscan1000	30	83 ± 10	< 0.001**	2 (7%)	60	82 ± 10	< 0.001**	2 (3%)
	Trios5	30	81 ± 10	< 0.001**	0 (0%)				
MSC	Neoscan1000	30	67 ± 7	< 0.001**	1 (3%)	60	72 ± 9	< 0.001**	2 (3%)
	Trios5	30	77 ± 8	< 0.001**	1 (3%)				
Total	Neoscan1000	150	86 ± 19	< 0.001*	16 (11%)	300	92 ± 22	< 0.001*	37 (12%)
	Trios5	150	97 ± 24		21 (14%)				

*Note:* Lower values indicate better usability (time) and feasibility (failures).

Abbreviations: CIC, conventional impression copings as scan body extensions; MSC, morphological scan body extensions positioned toward center of the arch; MSI, morphological scan body extensions positioned toward adjacent implant; NEX, control group without scan body extensions; NSK, Neoss ScanKey scan body extension.

* *p* < 0.05: Significant difference between scanners (Mann–Whitney *U*) or between scan body extension groups (Kruskal–Wallis).

** *p* < 0.0125: Significant post‐hoc difference from control (Kruskal–Wallis with Bonferroni adjustment).

In comparison to the control workflow, scan time was reduced by approximately 18%–41%, depending on the scan body extension technique. The largest time reduction was observed for both 3D‐printed scan body extension groups, whereas NSK showed a smaller but still significant time reduction.

### 3.5 Scan Failures

Two distinct failure patterns were observed during scanning. The first involved the algorithm misidentifying one scan body as another due to geometric similarity, causing the stitching to continue from a displaced position and generating a duplicated or misregistered arch (Figure 6A). The second involved minor movement of a nominally fixed structure, which introduced a parallel ghost model alongside the correct arch (Figure 6B). Both failure types require either extensive repairing or total rescanning to resolve.

**FIGURE 6:**
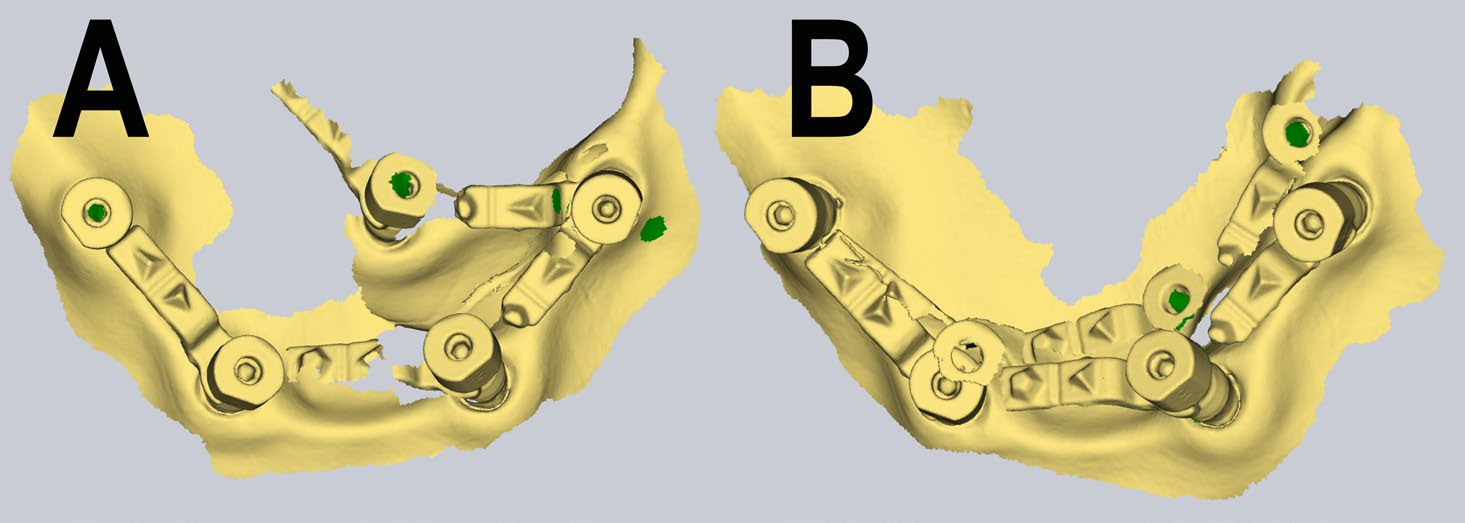
Representative examples of the two major scan failure types. (A) Algorithm confusion: Geometric similarity between scan body extensions caused the stitching algorithm to continue acquisition from an incorrect position, producing a displaced duplicate of the arch. (B) Movement‐induced failure: Minor displacement of a scan body extension during acquisition generated a parallel ghost model alongside the correct arch.

The overall frequency of major stitching errors resulting in failed scans was highest in the NSK group (Table 2). The use of other supplementary scan body extensions led to a relative reduction in scan failures, with the lowest failure rates observed for the MSI and MSC groups. Although failures were not eliminated entirely, all scan body extension techniques improved feasibility compared with control, with the effect being more pronounced for Trios5.

## 4 Discussion

The present in vitro study indicates that the addition of supplementary morphology can substantially improve usability, feasibility and accuracy, but that the magnitude and consistency of this improvement are influenced by both scan body extension design and the intraoral scanner used. These observations are consistent with prior work demonstrating that edentulous arches lack the distinct anatomical surface morphology necessary for accurate image stitching, leading to accumulated errors across long spans (Huang et al. 2020; Kernen et al. 2023). Huang et al. (2020) similarly reported that the major challenge in edentulous scanning is the limited number of stable morphological characteristics on the mucosa surface, resulting in large stitching errors. The present findings also align with those of Kernen et al. (2023), who observed that scan body extensions with irregular designs improved trueness compared to unsplinted scans, with values ranging from 26–181 μm versus 20–358 μm, respectively. Furthermore, the scanner‐dependent response observed in the present study corroborates the findings of Fu et al. (2025), who reported that prefabricated extensions yielded different levels of accuracy improvement depending on the intraoral scanner used, and Bader Eddin and Önöral (2024), who found that scan body extension design, scanner type, and their interaction all significantly influenced trueness outcomes.

A fundamental challenge in scan body extension design is that stitching algorithms are commercially protected and not publicly disclosed. Some scanners are known to prioritize tooth‐like structures and use subscans of approximately 15 × 15 to 20 × 20 mm, each containing on the order of 15,000–20,000 points. Grounded in the working principles described by Richert et al. and the finding by Kernen et al. (2022) that irregular geometry outperformed simpler designs, the custom scan body extensions were designed with morphological features comparable in size and complexity to natural tooth structures. Morphology was also randomly varied between scan body extensions to reduce the risk of the algorithm confusing one structure with another. This most likely also explains the lower failure rates observed with MSI and MSC compared with NSK and CIC, where simpler or repetitive geometries offered fewer distinguishing features for algorithmic matching. As algorithms remain proprietary, design optimization must rely on empirical testing, and the present study reflects this approach. The scanner‐dependent magnitude of improvement observed here aligns with findings by Fu et al. (2025) supporting the view that fundamental differences in image acquisition and stitching processing exist between devices.

Besides the effect on accuracy, the highly morphological scan body extensions also improved usability. The reduction in scan time observed for the MSI and MSC groups can be attributed to the decreased likelihood of the scanner losing track during acquisition. When tracking is lost, the operator must relocate to a previously scanned area for the software to re‐establish position; in featureless regions this recovery can be time‐consuming or may fail entirely (Richert et al. 2017). The unique, non‐repetitive morphology of the custom scan body extensions appears to facilitate faster recovery, as the algorithm can more readily identify a distinctive reference point.

A further critical design consideration was rigid fixation of the scan body extensions to the scan bodies. Secure mounting is essential, as even minor displacement during scanning, whether due to contact with the scanner tip or interaction with surrounding structures, can lead to complete scan failures. The high failure rate observed in the NSK group reflects an inherent characteristic of the manufacturer's intended attachment mechanism. As the scan body has a circular cross‐section and the ScanKey design incorporates no anti‐rotation feature, the extensions remained free to rotate after seating. Rotational displacement during acquisition is therefore a fundamental limitation of this design rather than an artifact of the printing or assembly process used in this study. A different resin material or tighter printing tolerances may improve friction between the ScanKey and the scan body; however, the very small contact area between the parts makes it unlikely that friction alone could provide sufficient rotational stability. This was difficult to avoid even under controlled in vitro conditions and would likely be exacerbated in a clinical setting. In contrast, the use of titanium mounting rings and screw fixation for the custom scan body extensions was intended to minimize unintended movement, and the substantially lower failure rates observed with the MSI and MSC groups suggest that this approach was largely effective. However, scan failures were not eliminated entirely, which may indicate that some residual rotational movement persists. The friction fit between the mounting screw and the scan body may not provide complete rotational stability. Future designs incorporating anti‐rotation features, such as indexed or keyed interfaces, may further improve fixation and reduce failure rates. In a clinical setting, the scan body extensions are intended to be assembled extraorally onto the scan bodies before intraoral placement, as the angulation and location of the fixation screws would frequently prevent intraoral access.

The failure data also revealed a scanner‐dependent pattern. For the NSK and CIC groups, Trios 5 showed notably higher failure rates than NeoScan1000 (37% versus 20% for NSK; 17% versus 10% for CIC). For the morphological designs, this pattern reversed or equalized, with Trios 5 recording 0% failures for MSI and 3% for MSC, compared to 7% and 3% for NeoScan1000 respectively. This suggests that Trios 5 may be more sensitive to geometric simplicity or repetitive geometry in the extensions, making it more susceptible to misregistration when distinguishing features are limited. Conversely, when provided with sufficiently complex morphology, Trios 5 appears to benefit more than NeoScan1000, as also reflected in its larger accuracy improvements for the morphological groups. As the stitching algorithms of both scanners are proprietary and undisclosed, the precise mechanisms underlying these differences cannot be determined from the present data, and this interpretation remains speculative.

The differences observed between the two laboratory scanner datasets, acquired before and after scan body reattachment, ranged from 12 to 24 μm. This is close to the stated accuracy tolerance of the laboratory scanner (3Shape E3, ISO 12836: 7 μm). While small relative to the IOS deviations observed across all experimental groups, this potential source of variability should be kept in mind when interpreting the results.

From a clinical perspective, the results obtained with Trios5 are particularly noteworthy. For both morphological scan body extension configurations, the deviations for the longest inter‐implant span were below 100 μm, which is within the misfit threshold previously defined as clinically acceptable for implant frameworks in the edentulous mandible (Andriessen et al. 2014; Kernen et al. 2022). While this does not guarantee passive fit, it suggests that highly morphological scan body extensions may bring digital complete‐arch impressions closer to clinically acceptable accuracy levels and represents a promising step toward more predictable digital workflows in edentulous implant cases.

Several limitations must be considered when interpreting these findings. The experimental model lacked the optical complexity of the intraoral environment, including saliva, soft tissue movement, and variable reflectivity. These factors may reduce the achievable benefits observed under controlled laboratory conditions. In particular, the centrally oriented morphological configuration (MSC), while effective in vitro, may be difficult to use clinically due to tongue interference. Although MSC showed marginally better precision, the inter‐implant design (MSI) produced the most consistent trueness improvements across both scanners and represents a more clinically accessible orientation. For clinicians translating these findings to practice, MSI is therefore the more viable starting point in mandibular cases, as its extensions project toward adjacent implants rather than into the center of the arch, reducing the risk of displacement by oral muscles.

The implant‐abutment connection surface in the experimental model was also positioned at the gingival margin, which deviates from the bone‐level position typically encountered clinically. In cases with bone‐level abutment platforms, the scan body extensions would sit in closer proximity to the gingival ridge, which may facilitate scanning by reducing the depth of the scanning field and minimizing the risk of the scanner losing focus between the extensions and the surrounding tissue. The present model therefore may not fully reproduce this aspect of the clinical scenario. In cases of deeper implant placement where extensions contact the gingival ridge, a pre‐operative scan acquired before extension placement would be required, consistent with the standard two‐stage edentulous implant scanning protocol.

Despite these limitations, the proposed approach has practical advantages. The scan body extensions can be produced using widely available SLA printers, making the method accessible to clinicians. The primary technical limitation lies in the requirement for secure mounting interfaces, such as the titanium rings used in this study, which currently require precision manufacturing.

A potential future development would be the integration of threaded interfaces directly into scan bodies by manufacturers, allowing scan body extensions to be rigidly attached without additional components. Alternatively, the mounting rings could be 3D‐printed as a single unit with the scan body extensions, eliminating the need for separate titanium components. However, this approach requires patient‐specific designs to accommodate the varying implant angulations encountered in each clinical case, thereby increasing design complexity and reducing the universality of the system. Such adaptations could nonetheless facilitate broader clinical adoption by reducing costs and simplifying chairside assembly.

Future research should focus on validating these findings under clinical conditions. The intraoral environment introduces challenges that may attenuate the benefits observed in vitro, making clinical studies in edentulous patients essential to determine whether the accuracy improvements translate into clinically acceptable framework fit. Furthermore, future investigations should examine a broader range of intraoral scanners to establish generalizability. Longitudinal clinical studies assessing prosthetic outcomes, such as screw loosening and framework fractures, would also help clarify whether the improved trueness achieved with morphological scan body extensions contributes to better long‐term clinical performance.

In conclusion, within the limitations of this in vitro study, highly morphological 3D‐printed scan body extensions significantly improved accuracy of complete‐arch digital implant impressions compared with an unmodified control workflow. The custom‐made scan body extensions also enhanced usability by reducing scan time and improved feasibility by lowering failure rates. These benefits were most pronounced for longer inter‐implant spans and when using the Trios5 scanner. While prefabricated scan body extensions and conventional impression copings offered more limited improvements, the morphological designs tested in this study represent a promising and accessible strategy for overcoming the inherent challenges of digital impressions in edentulous arches. Of the two morphological configurations, MSI represents the more clinically viable option, as its inter‐implant orientation reduces the risk of displacement by oral muscles.

## Author Contributions

**Ann Wennerberg:** supervision, resources, writing – review and editing, conceptualization. **Michael Braian:** conceptualization, methodology, software, supervision, validation, writing – review and editing. **Victoria Franke Stenport:** conceptualization, supervision, resources, funding acquisition, writing – review and editing, project administration. **Julian Hessel Baranowski:** conceptualization, methodology, investigation, validation, software, formal analysis, data curation, project administration, writing – review and editing, visualization, writing – original draft, funding acquisition.

## Funding

This research was supported by TUA Research Funding; The Sahlgrenska Academy at University of Gothenburg/Region Västra Götaland, Sweden and by the Public Dental Service (Folktandvården), Östergötland, Sweden. Neoss (Gothenburg, Sweden) provided implant analogs, multi‐unit abutments, scan bodies, and the NeoScan 1000 intraoral scanner used in this study.

## Ethics Statement

This study used a laboratory phantom model only. No human participants, human tissue, or patient data were involved at any stage. The study therefore falls outside the scope of research requiring ethics committee approval. The authors reviewed the reporting checklists listed in the journal's author guidelines and found none applicable to this in vitro accuracy study.

## Conflicts of Interest

The authors declare no conflicts of interest.

## Supporting information


**Table S3:** Descriptive statistics of absolute linear distance deviations (P1–P3) for trueness and precision across scan body extension groups and scanners. Lower values represent higher accuracy.

**Table S4:** Descriptive statistics of absolute linear distance deviations (P1–P2) for trueness and precision across scan body extension groups and scanners. Lower values represent higher accuracy.

## Data Availability

The data that support the findings of this study are available from the corresponding author upon reasonable request.
