# Supplementary material for: Investigations of Intraoral Scan Body Extensions. Effect of Design on the Accuracy, Usability, and Feasibility for Digital Full‐Arch Implant Scans In Vitro
Source: Clin Oral Implants Res. 2026 Jul 17;37(10):1172–83. doi: 10.1111/clr.70158 (PMC13644545; doi:10.1111/clr.70158)
Supplement: Supplementary file 1 [file CLR-37-1172-s001.docx]

| **Supplementary table 3. Descriptive statistics of absolute linear distance deviations (P1–P3) for  trueness and precision across scan body extension groups and scanners.** *Lower values represent higher accuracy.* | | | | | | | | | | | |
| --- | --- | --- | --- | --- | --- | --- | --- | --- | --- | --- | --- |
|  |  | **Across scan body extension groups split by scanner** | | | | | **Across scan body extension groups** | | | | |
| **Scan body extension group** |  |  | **Trueness** | | **Precision** | |  | **Trueness** | | **Precision** | |
|  | **Scanner** | **n** | **Mean (µm)** | **p-value** | **SD (µm)** | **p-value** | **n** | **Mean (µm)** | **p-value** | **SD (µm)** | **p-value** |
| NEX | Neoscan1000 | 30 | 160 | – | ± 29 | – | 60 | 122 | – | ± 51 | – |
|  | Trios5 | 30 | 85 | – | ± 39 | – |  |  |  |  |  |
| NSK | Neoscan1000 | 30 | 139 | 0.004** | ± 57 | 0.025 | 60 | 108 | 0.043 | ± 57 | 0.95 |
|  | Trios5 | 30 | 77 | 0.356 | ± 38 | 0.614 |  |  |  |  |  |
| CIC | Neoscan1000 | 30 | 115 | <0.001** | ± 34 | 0.524 | 60 | 82 | <0.001** | ± 51 | 0.91 |
|  | Trios5 | 30 | 49 | <0.001** | ± 43 | 0.695 |  |  |  |  |  |
| MSI | Neoscan1000 | 30 | 130 | 0.007** | ± 40 | 0.184 | 60 | 85 | <0.001** | ± 56 | 0.181 |
|  | Trios5 | 30 | 41 | <0.001** | ± 26 | 0.044 |  |  |  |  |  |
| MSC | Neoscan1000 | 30 | 104 | <0.001** | ± 27 | 0.582 | 60 | 91 | 0.001** | ± 33 | <0.001*** |
|  | Trios5 | 30 | 78 | 0.687 | ± 34 | 0.307 |  |  |  |  |  |
| Total | Neoscan1000 | 150 | 130 | <0.001* | ± 43 | 0.022* | 300 | 98 | <0.001* | ± 52 | <0.001*** |
|  | Trios5 | 150 | 66 | <0.001* | ± 40 | 0.212 |  |  |  |  |  |
| NEX – control group without scan body extensions; NSK – Neoss ScanKey scan body extension; CIC – conventional impression copings used as scan body extensions, MSI – morphological scan body extensions positioned towards adjacent implant; MSC – morphological scan body extensions positioned towards center of the arch | | | | | | | | | | | |
| *  *p < 0.05:* Significant difference between scanners (Mann–Whitney U) or between scan body extension groups (Kruskal–Wallis). ** *p < 0.0125:* Significant post-hoc difference from control (Kruskal–Wallis with Bonferroni adjustment). *** *p < 0.0125:* Significant variance difference from control (pairwise Levene’s tests with Bonferroni correction). | | | | | | | | | | | |
