# Supplementary material for: Investigations of Intraoral Scan Body Extensions. Effect of Design on the Accuracy, Usability, and Feasibility for Digital Full‐Arch Implant Scans In Vitro
Source: Clin Oral Implants Res. 2026 Jul 17;37(10):1172–83. doi: 10.1111/clr.70158 (PMC13644545; doi:10.1111/clr.70158)
Supplement: Supplementary file 2 [file CLR-37-1172-s002.docx]

| **Supplementary table 4. Descriptive statistics of absolute linear distance deviations (P1–P2) for  trueness and precision across scan body extension groups and scanners.** *Lower values represent higher accuracy.* | | | | | | | | | | | |
| --- | --- | --- | --- | --- | --- | --- | --- | --- | --- | --- | --- |
|  |  | **Across scan body extension groups split by scanner** | | | | | **Across scan body extension groups** | | | | |
| **Scan body extension group** |  |  | **Trueness** | | **Precision** | |  | **Trueness** | | **Precision** | |
|  | **Scanner** | **n** | **Mean (µm)** | **p-value** | **SD (µm)** | **p-value** | **n** | **Mean (µm)** | **p-value** | **SD (µm)** | **p-value** |
| NEX | Neoscan1000 | 30 | 109 | – | ± 25 | – | 60 | 67 | – | ± 49 | – |
|  | Trios5 | 30 | 25 | – | ± 25 | – |  |  |  |  |  |
| NSK | Neoscan1000 | 30 | 110 | 0.85 | ± 30 | 0.61 | 60 | 69 | 0.72 | ± 47 | 0.32 |
|  | Trios5 | 30 | 29 | 0.196 | ± 15 | 0.279 |  |  |  |  |  |
| CIC | Neoscan1000 | 30 | 84 | 0.002** | ± 33 | 0.036 | 60 | 66 | 0.60 | ± 35 | <0.001*** |
|  | Trios5 | 30 | 48 | <0.001** | ± 29 | 0.208 |  |  |  |  |  |
| MSI | Neoscan1000 | 30 | 62 | <0.001** | ± 17 | 0.161 | 60 | 51 | 0.21 | ± 23 | <0.001*** |
|  | Trios5 | 30 | 40 | 0.003** | ± 24 | 0.299 |  |  |  |  |  |
| MSC | Neoscan1000 | 30 | 61 | <0.001** | ± 19 | 0.361 | 60 | 47 | 0.04 | ± 25 | <0.001*** |
|  | Trios5 | 30 | 33 | 0.069 | ± 22 | 0.856 |  |  |  |  |  |
| Total | Neoscan1000 | 150 | 85 | <0.001* | ± 33 | 0.007* | 300 | 60 | 0.04 | ± 38 | <0.001*** |
|  | Trios5 | 150 | 35 | <0.001* | ± 25 | 0.078 |  |  |  |  |  |
| NEX – control group without scan body extensions; NSK – Neoss ScanKey scan body extension; CIC – conventional impression copings used as scan body extensions,  MSI – morphological scan body extensions positioned towards adjacent implant; MSC – morphological scan body extensions positioned towards center of the arch | | | | | | | | | | | |
| * p < 0.05: Significant trueness difference between scanners (Mann–Whitney U), between scan body extension groups (Kruskal–Wallis), or for precision (Levene's test). ** p < 0.0125: Significant post-hoc difference from control (Kruskal–Wallis with Bonferroni adjustment). *** p < 0.0125: Significant variance difference from control (pairwise Levene’s tests with Bonferroni correction). | | | | | | | | | | | |
